# The Effect of Pulsatile Flow on bMSC-Derived Endothelial-Like Cells in a Small-Sized Artificial Vessel Made by 3-Dimensional Bioprinting

**DOI:** 10.1155/2018/7823830

**Published:** 2018-04-17

**Authors:** Kang Woog Lee, Dae-Hyun Kim, Jun Hee Lee, Young-Nam Youn

**Affiliations:** ^1^Division of Cardiovascular Surgery, Severance Cardiovascular Hospital, Yonsei University College of Medicine, Seoul, Republic of Korea; ^2^Department of Nature-Inspired Nanoconvergence System, Korea Institute of Machinery and Materials (KIMM), Daejeon, Republic of Korea

## Abstract

Replacement of small-sized vessels is still challenging. This study is aimed at investigating the possibility of small-sized artificial vessels made by 3-dimensional bioprinting and the effect of pulsatile flow on bMSC-derived endothelial-like cells. Cells were harvested from rabbit bone marrow and primary cultured with or without growth factors. Endothelial differentiation was confirmed by the Matrigel tube formation assay, Western blot, and qRT-PCR. In addition, embedment of endothelial-like cells in an artificial vessel was made by 3-dimensional bioprinting, and the pulsatile flow was performed. For pumped and nonpumped groups, qRT-PCR was performed on CD31 and VE-cadherin gene expression. Endothelial-like cells showed increased gene expression of CD31 and VE-cadherin, and tube formation is observed at each week. Endothelial-like cells grow well in a small-sized artificial vessel made by 3-dimensional bioprinting and even express higher endothelial cell markers when they undergo pulsatile flow condition. Moreover, the pulsatile flow condition gives a positive effect for cell observation not only on the sodium alginate hydrogel layer but also on the luminal surface of the artificial vessel wall. We have developed an artificial vessel, which is a mixture of cells and carriers using a 3-dimensional bioprinting method, and applied pulsatile flow using a peristaltic pump, and we also demonstrated cell growth and differentiation into endothelial cells. This study suggests guidelines regarding a small-sized artificial vessel in the field of tissue engineering.

## 1. Introduction

Cardiovascular disease is a major cause of death [1]. Especially obstructive diseases in small-diameter (<6 mm) vessels, including coronary and peripheral artery vessels, are forming an ever-greater percentage of the death rate [2]. Arterial replacement or bypass grafting surgery is the treatment of choice for these obstructive diseases [3], and in the case of bypass surgery, autologous vascular grafts or artificial grafts are widely used recently. However, there are several disadvantages with autologous grafts such as the necessity of harvesting procedure and insufficient autologous graft length due to pathological change [4]. Moreover, it has also been reported that restenosis occurs at a high rate [5]. Therefore, artificial blood vessels are indispensable for solving these problems. In the case of middle-to-large vessels, an artificial graft made of ePTFE (expanded polytetrafluoroethylene) or PET (polyethylene terephthalate) is used to replace diseased arteries recently [6]. However, it is not recommended in small-diameter vessels, including coronary artery or genicular artery vessels, due to the high risk of restenosis [7].

For this reason, much research has been reported on overcoming the limitations of small-diameter vascular grafts. There are major approaches such as the use of artificial scaffold matrices made by electrospinning, freeze drying, or casting and cell seeding onto printed scaffold matrices [8–11]. However, it is not easy to design complex vascular structure, as the process of cell seeding requires time and there is difficulty in making artificial tissue composed of two or more cells. Recently, a three-dimensional bioprinting technique, which is a concept of printing complex artificial tissue composed of carriers and cells, has been introduced as a method for overcoming this problem [12, 13]. By applying this method, it is possible to shorten the time required for seeding cells by directly printing the cells on a scaffold, along with the advantage that various types of patient-specific artificial tissue can be produced well [14].

In this study, we developed a small-diameter artificial vessel composed of bone marrow-derived mesenchymal stem cells (bMSCs) in a sodium alginate hydrogel and polycaprolactone (PCL) scaffold using the three-dimensional bioprinting technique. In addition, we evaluate the effect of pulsatile flow on the differentiation of the bMSCs into the vascular endothelial cells.

## 2. Materials and Methods

### 2.1. Scheme of the Study Design and Animal Procedures

**Figure 1:**
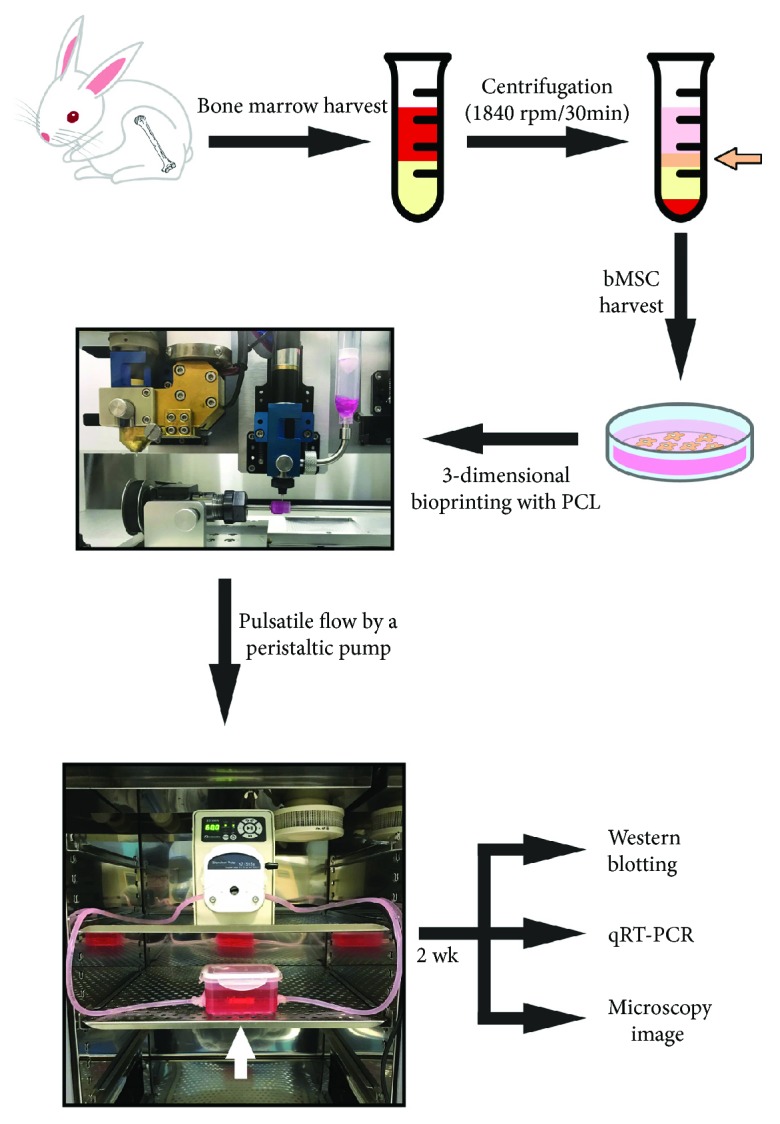
Scheme diagram of the experimental design.

A schematic diagram of the experiment is shown in Figure 1. The Yonsei University Health System Institutional Animal Care and Use Committee approved all animal procedures in this paper based on the guidelines for the care and use of laboratory animals (2015-0020).

### 2.2. Rabbit bMSC Isolation and Culture

A mature 16-week-old white male New Zealand rabbit weighing 3.5 kg was intramuscularly injected with 5 mg/kg xylazine and 10 mg/kg Zoletil® at 15 min intervals for anesthesia. Using a 13 G bone marrow biopsy needle (Angiotech Medical Device Technologies Inc., FL, USA), bone marrow (BM) was harvested from the femur and stored in a heparinized 50 mL conical tube (SPL Life Sciences, Gyeonggi-do, Korea) to inhibit coagulation. Then, it was filtered through a 40 *μ*m Cell Strainer (Life Sciences, NY, USA) and mixed with phosphate-buffered saline (PBS) up to 8 mL.

The MSCs were harvested following the previous study [15]. The BM-PBS mixture was centrifuged at 1500 rpm for 5 min; then, the supernatant was discarded and the remaining precipitate was suspended with 8 mL serum-free Dulbecco's modified Eagle's medium-low glucose (DMEM-LG) (Welgene, Daegu, Korea). The mixture was carefully transferred to a 15 mL conical tube (SPL Life Sciences, Gyeonggi-do, Korea) containing 6 mL of Ficoll-Paque® (Sigma-Aldrich, MO, USA) and then centrifuged at 1840 rpm for 30 min. After centrifugation, the interphase (cloud-like layer) was harvested and mixed with medium up to 10 mL per 15 mL conical tube. Then, it was centrifuged at 1500 rpm for 5 min, and the supernatant was removed. The cell pellet was mixed with DMEM-LG (Welgene, Daegu, Korea) supplemented with 10% fetal bovine serum (FBS) (GE Healthcare Life Sciences, PA, USA) and 1% penicillin-streptomycin (Thermo Fisher Scientific, MA, USA).

The culture medium was carefully changed after three days and then every two days thereafter. The culture was maintained at 37°C in a 5% CO_2_ incubator. After one week past, cells were subcultured and defined as primary-cultured mesenchymal stem cell passage 1.

### 2.3. Cell Differentiation into Endothelial-Like Cells

The bMSC was seeded into 100 mm dishes or 6-well plates at 100,000 cells/mL in DMEM-LG basal medium. At the next day, the culture media was discarded by aspiration and the cells were washed with PBS, followed by the addition of DMEM-LG (Welgene, Daegu, Korea) supplemented with 10% fetal bovine serum (FBS) (GE Healthcare Life Sciences, PA, USA), 1% penicillin-streptomycin (Thermo Fisher Scientific, MA, USA), 10 *μ*g/mL amphotericin B (Enzo Life Sciences, NY, USA), 50 *μ*g/mL gentamicin (Life Sciences, NY, USA), 50 *μ*g/mL vascular endothelial growth factor (VEGF) (Kingfisher Biotech, MN, USA), 10 ng/mL basic fibroblast growth factor (bFGF) (ProSpec, NJ, USA), and 1x ITS+3 solution (Sigma-Aldrich, MO, USA). Nonadded growth factor medium was prepared for use which is the negative control group.

### 2.4. Immunocytochemistry

Cells were cultured until passage 2, and 50,000 cells/mL were seeded on a poly-L-lysine-coated cover glass in a 6-well cell culture plate maintained at 37°C in a 5% CO_2_ incubator for 2 days in order to perform immunocytochemistry. Briefly, the cells were seeded into a poly-L-lysine-coated cover glass, fixed with ice-cold methanol for 10 min, and washed three times with PBS for 5 min each. Then, they were incubated with 1% BSA in PBS-T buffer for 30 min to block unspecific antibody binding. Cells were then incubated with each primary anti-mouse monoclonal antibody against CD34 diluted at 1 : 500 (Abcam, Cambridge, UK), CD44 diluted at 1 : 500 (Abcam, Cambridge, UK), *α*-smooth muscle actin (*α*-SMA) diluted at 1 : 1000 (Abcam, Cambridge, UK), and CD31 diluted at 1 : 1000 (Abcam, Cambridge, UK) in PBS-T with 1% BSA for 60 min at room temperature. Cells were then washed three times with PBS-T buffer and incubated with the secondary antibody, goat anti-mouse Cy3 ™ (Bethyl Laboratories, TX, USA), diluted at 1 : 500 for 60 min in a dark room to activate and preserve fluorescence. All microscopic images were created on an Olympus® DP71 microscope digital camera installed on an Olympus BX51TF system microscope (Olympus, Tokyo, Japan).

### 2.5. Tube Formation Assay

Cells were cultured on DMEM-LG growth medium for 4 weeks prior to the assay. The assay was initiated by coating a 24-well cell culture plate (SPL, Gyeonggi-do, Korea) with 150 mL of growth factor-reduced Matrigel (BD Biosciences, NJ, USA) per well. Approximately 50,000 cells were then seeded into each well and incubated at 37°C. After 12 hours later, light microscopic images were taken by the Olympus DP71 microscope digital camera installed on the Olympus BX51TF system microscope (Olympus, Tokyo, Japan).

### 2.6. Western Blot

Cells were seeded at 50,000 cells/mL in a 6-well cell culture plate and harvested every week from week 1 to week 4.

Protein was extracted from passage 2 cells with 500 *μ*L of PRO-PREP® protein extraction solution (containing 1.0 mM PMSF, 1.0 mM EDTA, 1 *μ*M pepstatin, 1 *μ*M leupeptin, and 1 *μ*M aprotinin) (Intron Biotechnology, Gyeonggi-do, Korea). All procedures were performed on ice. After 30 minutes, homogenized samples were centrifuged at 14,000 rpm at 4°C for 10 minutes, and the supernatant protein was extracted.

Quantification of the protein content was performed with the Protein-Assay Kit (Bio-Rad, CA, USA). For immunoblotting, each 20 *μ*g of total protein extract was mixed with Laemmli sample buffer and transferred to a Mini Protean® TGX™ precast gradient gel (Bio-Rad, CA, USA) with DOKDO-MARK™ (Intron Biotechnology, Gyeonggi-do, Korea) as a standard prestained protein marker. Gel electrophoresis was performed with a Mini Protean system (Bio-Rad, CA, USA) with 100 V applied for 130 minutes. After electrophoresis, proteins were blotted to a methanol-preactivated PVDF membrane, with 230 mAh applied for 90 minutes using a Mini Trans-Blot® Cell (Bio-Rad, CA, USA).

Blotted membranes were blocked for 60 minutes with PBS-T (phosphate-buffered saline containing 0.05% of Tween 20) with 5% bovine serum albumin (BSA). After washing three times with PBS-T, membranes were incubated at room temperature for 60 minutes with the primary anti-mouse monoclonal antibody against CD31 diluted at 1 : 500 (Abcam, Cambridge, UK) and the anti-goat monoclonal antibody against VE-cadherin diluted at 1 : 250 (Santa Cruz, TX, USA) in PBS-T with 5% BSA. *β*-Actin was used as a loading control by staining with a *β*-actin mouse monoclonal antibody at a dilution of 1 : 2000 (Abcam, Cambridge, UK) and then incubated using a Rocker (FINEPCR, Gyeonggi-do, Korea) for 60 minutes. After incubation with the first antibody, membranes were washed with PBS-T buffer three times and then incubated with the secondary goat anti-mouse IgG-HRP diluted at 1 : 2000 (Enzo Life Sciences, NY, USA) and mouse anti-goat IgG-HRP diluted at 1 : 2000 (Enzo Life Sciences, NY, USA) for 60 minutes at room. Membranes were then washed, and protein band detection was performed with the ECL solution kit (DAEILLAB Service, Seoul, Korea). Then, membranes were developed using ImageQuant™ LAS 4000 (GE Healthcare Life Sciences, PA, USA). The image was then analyzed with the pixel density analysis method using the ImageJ software (National Institute of Health, NY, USA).

### 2.7. Manufacturing the Artificial Vessel

As a supporting layer, polycaprolactone (PCL; MW = 45,000) (Sigma-Aldrich, MO, USA) was used to print the first- and third-layer structure, and medium-viscosity sodium alginate (Sigma-Aldrich, MO, USA) was used to print the second layer.

The point to be emphasized is that the second layer of the artificial vessel was fabricated by the harvested mesenchymal stem cells. Cells were trypsinized from the cell culture dish and then mixed with the basal medium with FBS for neutralizing trypsin. And then, cell suspension was mixed with sterile sodium alginate gel powder. For detail, sterilized sodium alginate powder was mixed with cell suspension and then mixed with a low concentration of calcium chloride solution to increase viscosity. Then, we put this mixture into a dispenser for second-layer printing. After all printing processes, the artificial blood vessels were soaked to calcium chloride solution, so that the second layer could maintain its shape well due to its increased viscosity.

The first layer was fabricated in a diagonal cross-striped form for smooth exchange of growth medium materials. PCL was placed in a three-dimensional printing device (KIMM&Protek Korea, Daejeon, Korea) and dispensed through a 300 *μ*m nozzle at a temperature of over 100°C by a pneumatic pressure of 400 kPa.

The feed rate was 200 mm/min. The second layer was fabricated in a helical form, in which alginate gel with cells was placed in another channel of the same printing device and dispensed through a 400 *μ*m nozzle at a room temperature by a pneumatic pressure of 100 kPa. The feed rate was 300 mm/min. 1% of calcium chloride solution was used for enforced viscosity of sodium alginate hydrogel and 5% of calcium chloride solution for gelation of bioprinted sodium alginate gel.

The condition of the third layer was the same as that of the first layer but was fabricated in a helical form as in the cell-adherent layer. The major dimensions of the artificial vessel design were a length of 40 mm, an outer diameter of 8 mm, and an inner diameter of 5 mm.

A fabricated artificial vessel was then imaged using a field emission scanning electron microscope with a backscattered electron image detector and an environmental secondary electron detector (JEOL Ltd., Tokyo, Japan).

### 2.8. Simulated Blood Circulation System

Aseptic silicon tubing (ID = 3.1 mm) (Baoding Shenchen Precision Pump, Baoding City, China) was used for extracorporeal tubing, connected with a handcraft cell culture chamber. All components were sterilized with ethylene oxide gas, and a bioprinted artificial vessel was installed in the clean bench. Then, this entire set was installed into the peristaltic pump system (Baoding Shenchen Precision Pump, Baoding City, China) for creating a pulsatile flow. The perfusion rate was 60 times a minute and the perfusion pressure was about 40 mmHg, which are similar to those in the previous study [11]. Two nonpumped models were additionally prepared: one as a control group and another as a nonpumped and nonadded growth factor group (another control group). All cultures were added antibiotics every 3 days considering the half-life of the drug.

### 2.9. Live/Dead Assay

The pumped artificial vessel was soaked with a Live/Dead cell staining kit (Enzo Life Sciences, NY, USA), composed of Live-Dye™ and propidium iodide (PI), based on the permeability of cell membrane integrity. Microscopic images were obtained at a random area with a fluorescence image using the Olympus DP71 microscope digital camera installed on the Olympus BX51TF system microscope (Olympus, Tokyo, Japan). The image was then merged with the color channel merge method using the ImageJ software (National Institute of Health, NY, USA).

### 2.10. Real-Time Reverse Transcriptase Polymerase Chain Reaction (RT-PCR)

After 2 weeks of in vitro cell culture and tube circulation, cells were harvested using low-concentration EDTA solution in a 37°C environment for weakening the alginate bond [16]. It acts to lower the viscosity of the alginate gel. After that, cells were obtained by centrifugation and harvested. Total cellular RNA was extracted, and 1 *μ*g of cellular RNA was reverse-transcribed into complimentary DNA (cDNA) using a PrimeScript RT reagent kit (Takara, Shiga, Japan). Real-time quantitative RT-PCR primers targeting CD31 (forward: 5′ AGAGACGGTCTTGTCGCAGT 3′ and reverse: 5′ TACTGGGCTTCGAGAGCAGT 3′), VE-cadherin (forward: 5′ TCCTCTGCATCCTCACTATCACA 3′ and reverse: 5′ GTAAGTGACCAACTGCTCGTGAAT 3′), and *β*-actin (forward: 5′ GCTATTTGGCGCTGGACTT 3′ and reverse: 5′ GCGGCTCGTAGCTCTTCTC 3′) were prepared by oligonucleotide synthesis service (Bioneer Co., Daejeon, Korea).

The SYBR Premix Ex Taq™ (Tli RNase H Plus) (Takara, Shiga, Japan) and Real-time RT-PCR Detection System (Thermo Fisher Scientific, MA, USA) were used for detecting real-time quantitative PCR products from reverse-transcribed cDNA. The Ct value is the cycle number at which the fluorescence signal reaches a threshold.

The ∆Ct is determined by subtracting the Ct of the *β*-actin control from the Ct of the target gene as change of gene expression (∆Ct = Ct_target_ – Ct_*β*-actin_). To determine the ∆∆Ct value, it is calculated as the subtraction of the experimental group ∆Ct value from the control group ∆Ct value (∆∆Ct = ∆Ct_Experimental_ – ∆Ct_Control_). The relative value of target genes to the endogenous reference is described as the fold of *β*-actin = 2^−(∆∆Ct)^.

### 2.11. Confocal Microscopy

An artificial vessel was pumped for 2 weeks into the peristaltic pump system. After 2 weeks, the artificial vessel was sectioned by a surgical blade; then, the artificial vessel was embedded into the Live/Dead cell assay kit composed of Live-Dye and propidium iodide (PI). Then, immunofluorescence and bright-field counter images were visualized using a confocal microscope LSM 700 and compiled with a ZEN 2009 microscope and imaging software (Carl Zeiss GmbH, Oberkochen, Germany).

### 2.12. Statistical Analysis

PRISM 5 (GraphPad, CA, USA) was used for statistical analysis. Quantitative data are expressed as mean ± standard deviation (SD). Comparisons between quantitative data were performed using one-way analysis of variance (ANOVA). If significance was achieved in one-way analysis, post-ANOVA comparison of the means was performed using Tukey's multiple comparison test. Statistical significance for all analyses was defined as *P* < 0.05.

## 3. Results

### 3.1. Primary Cell Culture and Validation

**Figure 2:**
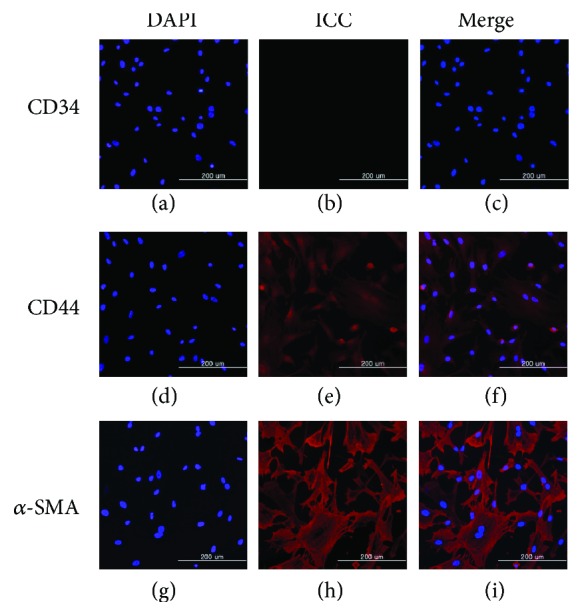
bMSC surface marker expression and validation by immunocytochemistry. The bMSCs were negative in CD34 (c) and positive in CD44 (f) and alpha-SMA (i). The scale bar indicates 200 *μ*m.

To define harvested cells as bMSCs, cells were cultured until passage 2. In the immunohistochemistry results, cells expressed CD44 protein as a major marker of stem cells but not expressed CD34 protein. Moreover, *α*-SMA was strongly expressed as a structure protein (Figure 2). Cell morphology also has a spindle shape. This is similar to the general shape of mesenchymal stem cells (Figures 2(g) and 2(h)).

### 3.2. Effect of Growth Factors on Protein Expression

**Figure 3:**
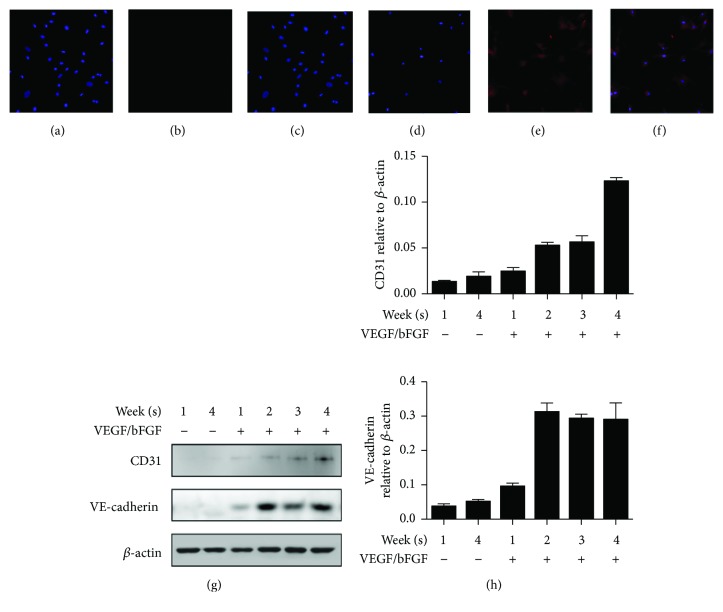
Effects of growth factor on endothelial cell-related protein expression by immunocytochemistry and Western blotting. The endothelial-like cells were negative in CD31 after 1 week (a–c) and positive in 1 month (d–f). The protein levels of CD31 and VE-cadherin increased in the growth medium (g). The mRNA expression levels of CD31 and VE-cadherin increased in the growth medium.

CD31 and VE-cadherin as endothelial cell markers and *β*-actin as a housekeeping gene were detected using immunocytochemistry and Western blot analysis. Endothelial-like cells cultured with DMEM-LG growth medium during 1 week did not express CD31 protein (Figures 3(a)–3(c)), but after 28 days, cells strongly expressed CD31 protein at the cell surface (Figures 3(d)–3(f)). As demonstrated by the Western blotting image, there were no significant CD31 and VE-cadherin protein expression changes in week 1 and 4 cultures in the basal medium group and week 1 cultures in the growth factor medium group (Figures 3(g) and 3(h)). In the growth factor medium group at week 2 to 4, CD31 and VE-cadherin were increased as the week progressed (Figures 3(g) and 3(h)).

### 3.3. Tube Formation Assay

**Figure 4:**
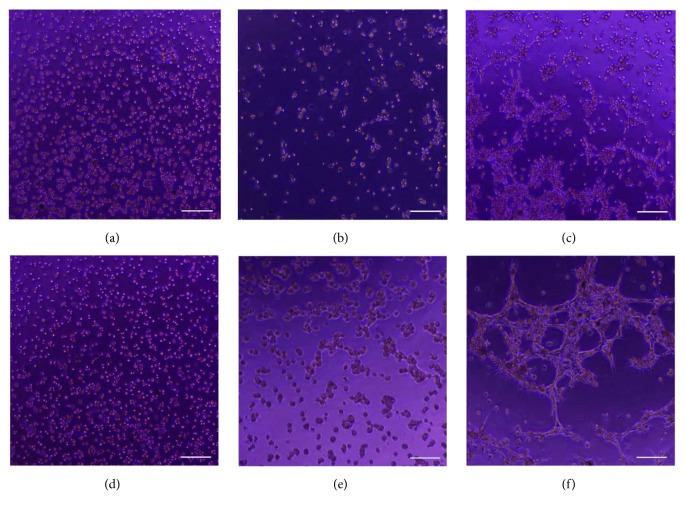
Endothelial-like cell tube formation assay. The chondrocytes after 6 hours (a) and 12 hours (d), the bMSCs after 6 hours (b) and 12 hours (e), and the endothelial-like cells after 6 hours (c) and 12 hours (f). There are tube formations at endothelial-like cells after 12 hours (f). The scale bar indicates 500 *μ*m.

To confirm the endothelial-like differentiation of bMSCs, the Matrigel-based tube formation assay was performed. Rabbit auricular cartilage cells did not show any interaction between cells after 6 and 12 hours (Figures 4(a) and 4(d)), and bMSCs showed a weak aggregation but did not have pseudopodia (Figures 4(b) and 4(e)). However, differentiated endothelial-like cells showed tube formation at light microscopic imaging where only endothelial cells are formed (Figures 4(c) and 4(f)).

### 3.4. Artificial Vessel Fabrication and Circulation

**Figure 5:**
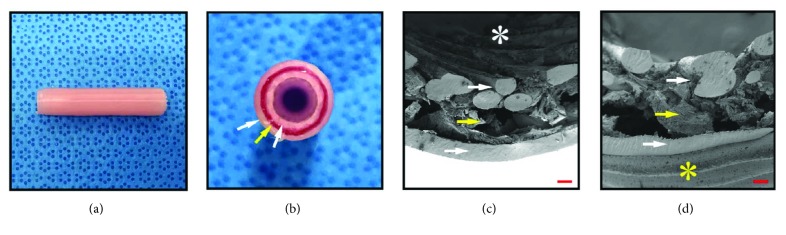
Gross and scanning electron microscope images of the artificial vessel; (a) longitudinal view, (b) transectional view, (c) SEM image of the inner surface, and (d) SEM image of the outer surface. The white arrow indicates the polycaprolactone layer, and the yellow arrow indicates the dried sodium alginate hydrogel layer due to SEM sampling. The white asterisk indicates the inner side surface woven in a cross-striped form, and the yellow asterisk indicates the outer surface woven in a helical form. The scale bar indicates 200 *μ*m.

To exchange the various blood factors, the first layer was fabricated in a striped pattern while the second and third layers were fabricated in a helix form (Figure 5). The fabricated artificial vessel was then soaked in a 5% calcium chloride solution. The completed artificial vessel had a pale pink color because of phenol red in the basal media (Figures 5(a) and 5(b)). The approximate dimensions of the artificial vessel were a length of 40 mm, an outer diameter of 8 mm, and an inner diameter of 5 mm, almost the same as we designed (Figures 5(a) and 5(b)), and each designed layer was positioned well (Figures 5(c) and 5(d)). There were no special problems such as contamination of breakdown of the artificial vessels during the pulsatile pump operation period.

### 3.5. Live/Dead Assay

**Figure 6:**
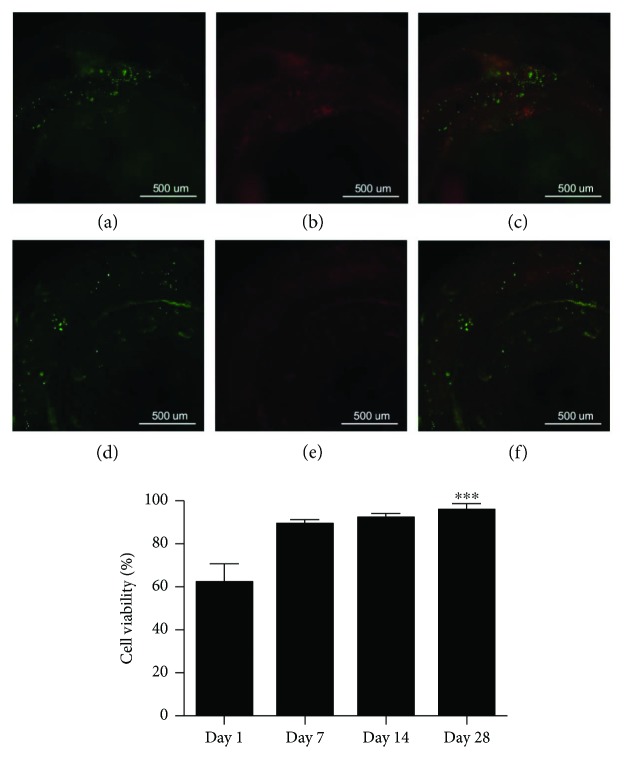
Time course of the live cell ratio using the Live/Dead assay. The pumped tube at day 1 had some dead cells (red) (a–c) while these dead cells decreased by day 28 (d–f). Cell viability was increased during day 1 to day 7 periods. Error bars indicate the SD. ^∗∗∗^*P* < 0.001.

The Live/Dead assay was performed every 7 days for 1 month, and Figure 6 shows the photographed fluorescence microscopic image along with the statistical graph. A cell-permeable green fluorescence Live-Dye was utilized to stain live cells, and dead cells were detected by the red fluorescence produced when PI binds to DNA. Live cells can exclude PI, so live cells expressed green fluorescence while dead cells expressed red fluorescence. We counted live and dead cells in all the pictures and divided them into total cells. The cell viability is calculated as live cells divided by total cells.

Microscopic observation at days 1, 7, 14, and 28 showed that dead cells significantly decrease but live cells had increased as days passed (Figures 6(a)–6(c) and 6(d)–6(f)). The live cell ratio had significantly increased from day 1 to day 7, but there was no significant difference among day 7 to day 28 (Figure 6).

### 3.6. Gene Expression

**Figure 7:**
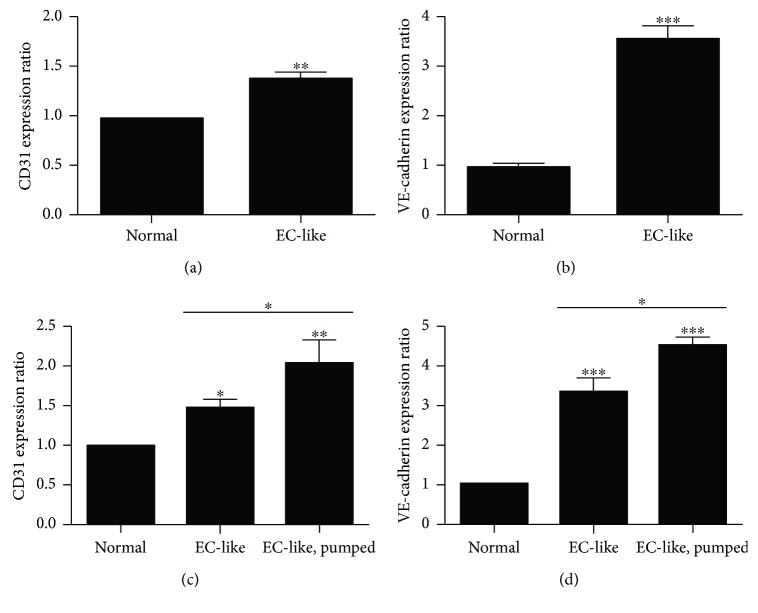
Vascular endothelial cell-related gene expression profile by qRT-PCR. The mRNA expression levels of CD31 in cells (a) and tubes (c). The mRNA expression levels of VE-cadherin in cells (b) and tube (d). Error bars indicate the SD. ^∗∗∗^*P* < 0.001, ^∗∗^*P* < 0.01, and ^∗^*P* < 0.05.

The mRNA expression levels of CD31 and VE-cadherin had significant differences between the basal medium group and the growth factor medium group. Cultures grown in the growth factor medium group expressed about 1.5-fold of CD31 mRNA expression level (Figure 7(a)) and 3.5-fold of VE-cadherin mRNA expression level (Figure 7(b)) compared with those grown in the basal medium group.

Moreover, the mRNA levels of CD31 and VE-cadherin in the peristaltic pumped group were highly expressed compared to those in the nonpumped group. The peristaltic pumped growth factor medium group expressed about 1.44-fold of CD31 mRNA expression level (Figure 7(c)) and 1.35-fold of VE-cadherin mRNA expression level (Figure 7(d)) compared with the nonpumped growth factor medium group.

### 3.7. Endothelial-Like Cell Engraftment into the Artificial Vessel Lumen

**Figure 8:**
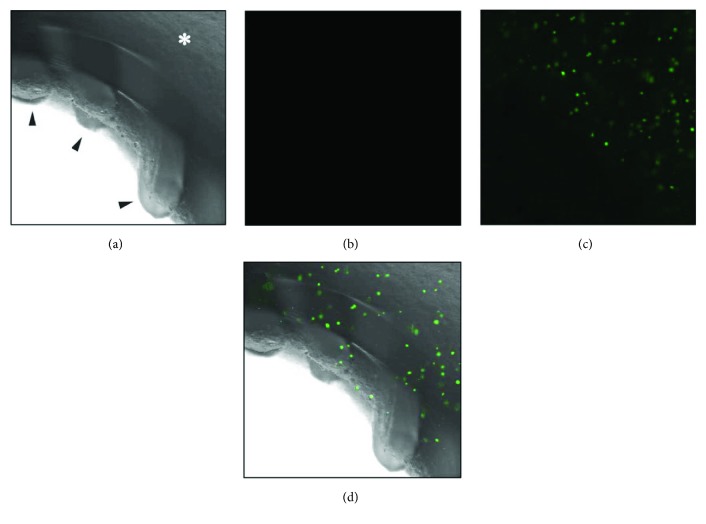
Confocal microscopic image of endothelial-like cell engraftment into the artificial vessel lumen. The inner side of the pumped tube had PCL structures in a cross-striped pattern (black head arrow). There are some live cells (green) (c) and no dead cells (red) (b) in the sodium alginate hydrogel layer (white asterisk). Live cells had engrafted into the lumen (d).

There are several points that live endothelial cells invade the artificial vessel lumen despite the pulsatile flow (Figure 8). In the phase-contrast bright-field image, sodium alginate gel did not wash out and even cells are all alive while no dead cells were observed (Figures 8(a) and 8(b)). The morphology of live cells has an irregular shape because of their adhesion and proliferation (Figures 8(c) and 8(d)).

## 4. Discussion

In this study, we made a small-diameter artificial vessel using the three-dimensional bioprinting technique with endothelial-like cells and polycaprolactone. Also, we demonstrated that the pulsatile flow promotes the expressions of vascular endothelial cell markers.

In previous research, nontoxic biodegradable materials are recommended when manufacturing small-sized artificial vessels [17]. Also, the materials should be biodegradable for tissue healing or for the regeneration process to prevent thrombosis or atherosclerosis [18]. The nondegradable materials, such as ePTFE and polyethylene terephthalate grafts, are widely used in vascular replacement surgery. However, they have limitations when used in small-diameter vessels because they could generate blood clots [6]. PCL is one of the commonly tried biodegradable materials in recent studies [11, 19, 20]. PCL degrades slowly because of hydrolysis of ester linkage, with the elimination of the resultant fragments by macrophages and giant cells, so there are no residues left [6]. Also, there is a previous study about a rabbit carotid grafting model using only the PCL artificial vessel graft [21]. Systolic pressure in adult rabbits ranged from 110 to 140 mmHg, while the diastolic pressure was from 95 to 120 mmHg [22], similar to the human blood pressure profile. Thus, our artificial blood vessel could also withstand the arterial pressure.

Direct cell seeding and encapsulation of the cells with a hydrogel are typical methods in the tissue engineering field [11, 23, 24]. Sodium alginate hydrogel has some advantages such as easy handling, gelation taking place at normal room temperature, and the fact that gelation can be more controlled by the cross-linker, calcium chloride solution. In addition, sodium alginate has neutral pH and does not react with cellular proteins [25, 26]. In this study, we confirmed that the number of living cells increased and the number of dead cells decreased during the experimental period in the cell bioprinting layer made of sodium alginate hydrogel.

Mesenchymal stem cells have been widely used for therapeutic purpose or cell-based tissue engineering because of their multipotent potential [11, 27, 28]. Moreover, mesenchymal stem cells could be harvested at various sources such as adipose tissue [29], umbilical cord [30], umbilical cord blood [31], and mainly bone marrow [11, 15, 32–34]. There are several studies confirming that bMSCs could be used in vascular regeneration purpose, because stem cells could be differentiated into vascular endothelial progenitor cells when stimulated by endothelial cell-secreted cytokine and they could be involved in vascular restoration and neovascularization [35, 36].

Cell markers are the keystones of identifying mesenchymal stem cells. Researchers have proved endothelial-like cell differentiation through molecular biology experiments due to the expression of endothelial cell markers such as CD31 or VE-cadherin [11] and confirmed the morphological characteristic through the tube formation assay [37, 38]. We used vascular endothelial growth factor and basic fibroblast growth factor according to previous studies [36, 39] and verified differentiated cells as endothelial-like cells. Moreover, we also confirmed that the pulsatile flow could more strongly induce endothelial cell-related marker expression in endothelial-like cells differentiated from mesenchymal stem cells.

The simulated blood circulation system was referred from existing studies. Researchers suggest various requirements such as constant perfusion pressure, incubator condition [11], flow chamber, and silicone tubing [40]. Also, researchers have reported that preconditioning, which is similar to in vivo after in vitro transplantation, facilitates the engraftment of the seeded or bioprinted cells [41, 42]. Our pulsatile flow system operated no leakage and no contamination during the experimental period, and the pumped tube underwent shear stress and pulsatile flow to show higher expression of CD31 and VE-cadherin as endothelial cell markers as described in a previous study [11]. This means that the artificial vessel in this study differentiated more endothelial-like cells in the pulsatile flow condition, and it is meaningful that we have mimicked the native vascular system, which has regular pressure and flow [43], which is the aim of tissue engineering [44].

In addition, in the course of this circulation, the artificial blood vessel did not lose sodium alginate hydrogel and the percentage of live cells was increased. It was confirmed that the pulsatile flow gave a positive effect on the survival of endothelial-like cells in the artificial blood vessel. Therefore, it is expected to have a similar result even when the artificial vessel graft is in the body.

As a result, the present study confirmed that growth factors affect the differentiation from MSCs into EC-like cells and pulsatile flow makes an additional positive effect to express endothelial cell markers such as CD31 and VE-cadherin in both cellular mRNA levels and protein levels. Moreover, we demonstrated that pulsatile flow could fabricate and influence the formation of the lumen consisting of differentiated EC-like cells. In particular, the strength of our research is that the artificial vessel produced using only a scaffold without cells showed vascular cell invasion where the artificial vessel invades the natural blood vessel, but our artificial blood vessel is able to keep cells alive due to the presence of hydrogel, which could show endothelial-like cell invasion entirely. This means that it can be expected to prevent the formation of blood clots due to endothelial-like cells in artificial vessels and to biodegrade PCL at an appropriate time. Moreover, recent studies have reported that the scaffold with stem cells expressing endothelial cell markers had a positive effect on long-term artificial vessel patency and vascular endothelial cell growth [45, 46].

One limitation of this study is that the endothelial cells were not completely covered into the artificial vessel lumen for 4 weeks. In other words, the period of 1 to 4 weeks may not be enough time to align cells to the lumen of the artificial vessel. However, according to a previous study, PCL grafts revealed an endothelialization of the luminal surface of the graft, spreading from the adjacent native aorta toward the graft body with a confluent monolayer of endothelial cells at 12 weeks [47]. There is another limitation. We tried to perform Western blot analysis both during cell culture and after artificial vessel circulation. Although the alginate gel dissolved using low-concentration EDTA solution, some alginate protein remained. So it was difficult to quantify the protein amount using the Bradford assay after sample harvest. Also, the absolute quantity of the target protein was small. So we had to perform only real-time RT-PCR.

We therefore expect that our artificial vessel would be covered faster due to endothelial-like cells in the sodium alginate hydrogel layer. Moreover, we also expect that the differentiated endothelial-like cells could be helpful to locate the vasa vasorum. Also, the present study is a preliminary study. We focused on the printing method which does not affect the cell viability, and we just emphasized that bone marrow-derived stem cells in the printed artificial vessel could differentiate into vascular endothelial-like cells in vivo. So further studies are needed; we are planning to conduct animal experiments in the near future.

## 5. Conclusion

In conclusion, we suggest the effective fabrication method of a small-sized artificial vessel. This paper will be helpful for various future studies, including those investigating tissue engineering of artificial organs. We are currently in the process of improvement and optimization to create more ideal artificial vessels.
